# Understanding the sensory and physicochemical differences between commercially produced non-alcoholic lagers, and their influence on consumer liking

**DOI:** 10.1016/j.fochx.2021.100114

**Published:** 2021-01-08

**Authors:** Imogen Ramsey, Qian Yang, Ian Fisk, Rebecca Ford

**Affiliations:** a Sensory Science Centre, Division of Food, Nutrition and Dietetics, School of Biosciences, University of Nottingham, Sutton Bonington Campus, Loughborough LE12 5RD, United Kingdom; b Samworth Flavour Laboratory, Division of Food, Nutrition and Dietetics, School of Biosciences, University of Nottingham, Sutton Bonington Campus, Loughborough LE12 5RD, United Kingdom

**Keywords:** Volatiles, Non-volatiles, Sensory, Consumer, Beer, Physicochemical

## Abstract

This study aimed to investigate the sensory and physicochemical differences of a range of commercial non-alcoholic lagers, as well as their influence on overall liking. Using physicochemical analysis and modified quantitative descriptive analysis (QDA) with a trained panel (n = 10) eighteen commercial non-alcoholic lagers, made using different production methods, were assessed. A subset (eleven), representing the sensory space were also assessed for hedonic liking using consumers (n = 104). Overall, it showed a clear variety of non-alcoholic lagers were selected, with different clusters of samples found with identifiable characteristics. Production methods were explored as a possible explanation for the differences in characteristics, however these did not fully explain the clusters and therefore other factors, such as pre or post processing methods are discussed. In terms of overall liking, five clusters of consumers were discovered with different patterns of liking, confirming that a wide range of non-alcoholic lagers are needed to satisfy all consumers.

## Highlights

• Variation in sensory and physicochemical profiles not explained by production method.
• Differences instead were discussed to be due to pre and post processing methods.
• Overall consumer liking could be optimised by mixing different production techniques.
• Five patterns of consumer liking identified, related to sensory characteristics.

## 1 Introduction

The international non-alcoholic beer (NAB) market is predicted to experience a rise in total volume growth of 24% by 2021 and be worth over $25bil by 2024 (Verma & Rawat, 2018), showing its value and importance in the drinks sector (Euromonitor, 2017). Interest in these products in the Middle East, Africa and Western Europe appear to be the drivers of this growth, with countries such as Germany owning 14% of the worldwide non-alcoholic drinks market (Euromonitor, 2017).

This increase in value is down to many factors, with 47% of consumers limiting their alcohol consumption compared to 12 months earlier (Mintel, 2019) and an increased drive from global manufacturers to emphasise responsible drinking (ABInBev, 2018). These factors have led to the consumer moderating their alcohol consumption, focusing on improving health, weight management and saving money (Mintel, 2017). The biggest challenge for breweries is to produce lower alcohol variants which taste more like their standard strength equivalents, with one in three consumers claiming this would sway them to drink more of these products (Mintel, 2017). Therefore, an opportunity has arisen for the growth of the low and non-alcoholic drinks sector, leading to an increase in the development of lower alcohol alternatives. One of the most interesting developments in this ever changing field is the introduction of craft breweries solely focusing on the production of low alcohol/NABs (Euromonitor, 2017), resulting in increased experimentation, innovation and development. Much of this innovation focuses on different production methods to produce appealing sensory profiles (Euromonitor, 2017).

**Fig. 1:**
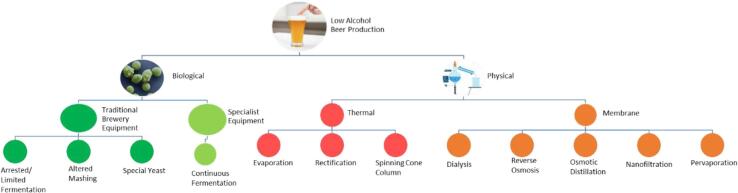
Non-alcoholic beer production methods. Green indicates biological methods, including traditional brewery equipment and specialist equipment. Physical methods are also shown, with red indicating thermal based methods and orange indicating membrane based technologies. (For interpretation of the references to colour in this figure legend, the reader is referred to the web version of this article.)

The production of NABs can be divided into two main categories: biological and physical methods. Biological methods focus on limiting ethanol production early on in the process, whilst physical methods remove ethanol post brewing. Different techniques are summarised in Fig. 1, with comprehensive reviews provided by Brányik et al., 2012, Bellut and Arendt, 2019. Biological methods can be split into those that use traditional brewing equipment (arrested or limited fermentation, altered mashing and special yeasts) and those that need specialist equipment (continuous fermentation). Previous studies have suggested that these techniques can cause decreases of up to 87% for esters and 80% for higher alcohols in comparison to original beers (Narziß, Miedaner, Kern, & Leibhard, 1992), resulting in a disharmonious final beer product, with wort-like off flavours and increased sweetness (Sohrabvandi, Mousavi, Razavi, Mortazavian, & Rezaei, 2010). However, there has been limited sensorial research characterising these properties. Detailed reviews on the physical methods of creating NABs, including industrial scale thermal based processes, such as spinning cone column (SSC) and vacuum distillation, have shown acceptable final products with reduced thermal stress (Brányik et al., 2012, Müller et al., 2017, Zufall and Wackerbauer, 2000). However, studies comparing the losses of volatiles by these methods found up to 100% of esters and up to 98% higher alcohols were lost in comparison to the original beer (Brányik et al., 2012, Zufall and Wackerbauer, 2000). Membrane processes include; dialysis, reverse osmosis (RO), osmotic distillation (OD), nanofiltration (NF) and pervaporation. To the authors knowledge, only two of these processes (dialysis and RO) are used on an industrial scale (Branyik et al., 2012) yet still result in large reductions in esters (up to 87%) and higher alcohols (up to 81%) (Kavanagh et al., 1991, Stein, 1993). The sensory properties of NABs made by both thermal and membrane based processes have resulted in beers described as having less aroma and body and more acidity (Montanari, Marconi, Mayer, & Fantozzi, 2009). To counteract this, some breweries have attempted to combine both biological and physical methods to produce a more sensorially acceptable NAB (Jiang et al., 2017).

The production method chosen to produce a NAB has previously been shown to impact the sensory qualities of beer (Krebs et al., 2018, Schmelzle et al., 2013). Research by Schmelzle, Lindemann, and Methner (2013) used descriptive analysis with semi-trained consumers to describe sensory differences amongst twelve samples produced through different techniques and they were able to divide them into ‘physical’ and ‘biological and mixed methods’. In another study, the impact of production technique on the macromolecular profile of commercial NABs was studied (Krebs, Müller, Becker, & Gastl, 2018) but only mouthfeel sensory descriptors and physical instrumental information was provided. Due to technological advances and the combining of production methods, further research is required to investigate the sensory and physicochemical impact of a wide range of production techniques that are currently being used within the brewing industry. Whilst several studies have investigated the loss of volatile compounds using different production techniques (Bellut and Arendt, 2019, Müller et al., 2017), only one has looked at the effect on the sensory properties of beer, as well as on consumer liking (Schmelzle, Lindemann, & Methner, 2013), which is critical for the brewing industry. The relationship between sensory characterisation and flavour chemistry would further advance knowledge regarding production of NAB, therefore guiding breweries towards practices they can use to improve the quality and consumer liking of their products.

The objectives of this study were therefore to investigate the physicochemical and sensorial properties of a range of commercially produced non-alcoholic lager style beers. This was achieved through developing a robust category wide non-alcoholic lager sensory lexicon using a trained sensory panel, whilst also correlating sensory data with physicochemical properties to reveal relationships for the wider category. Beers were clustered to understand similarities and differences, and possible effects of production method were explored to ascertain whether they had an effect on the overall characteristics of the beer, or whether other parameters were the source of these differences. Finally, the influence of these sensory properties on consumer liking were assessed.

## 2 Materials and methods

### 2.1 Samples

A range of non-alcoholic commercial lagers (n = 18) from the EU market were carefully selected to include a wide range of flavour characteristics and production methods (discovered by either intellectual property or from brand websites). The production methods were split into five categories, which included: altered brewing, special yeasts, dealcoholized (samples that used thermal or membrane based technologies), vacuum distillation and mixed methods (samples that underwent both biological and physical processing). Details are shown in Table 1. Samples were kept in cold storage at 4 ± 2 °C before assessments commenced.

**Table 1:** Beer samples, production methods, size of brewery, additional ingredients and physicochemical analysis results. Size of brewery is described as either M (multinational brewery) or C (craft brewery). Additional ingredients were those described on commercial beer labels, which included anything other than water, barley malt, yeast and hops. Different letters within a column^abc^ represent a significant difference among samples in terms of physicochemical parameters (Tukeys HSD, p < 0.05). Samples with an asterisk (*) were those selected for the subset for consumer overall liking sensory analysis.

Sample Number	Production Method	Size of Brewery	Additional Ingredients	Ethanol Content (ABV)	pH	Bitterness units (BU)	Total Polyphenols (mg/L)	Fermentable Sugars (g/L)				
								Glucose	Sucrose	Fructose	Maltose	Maltotriose
1*	Altered Brewing	M	Wheat	0.05^efg^	4.48^c^	18.29^b^	49.20^h^	2.79^abc^	0.20^c^	0.86^cde^	12.64^a^	5.13^a^
2*	Altered Brewing	C	Rye, Wheat, Maltodextrin	0.57^b^	4.81^a^	17.38^bc^	114.80^cd^	0.04^f^	0.01^c^	0.02^g^	0.00^e^	1.41^cde^
3*	Altered Brewing	M	Corn	0.03^g^	4.44^cde^	13.68^defg^	114.16^cd^	1.69^cde^	0.19^c^	0.91^cd^	9.35^abcd^	3.39^abc^
4	Altered Brewing	M	Flavouring	0.12^e^	4.14^gh^	15.44^cd^	115.71^cd^	3.04^ab^	0.30^bc^	1.17^c^	10.17^abc^	3.71^abc^
5*	Special Yeast	M	Modified hop products	0.06^efg^	4.41^de^	12.49^fg^	119.90^c^	3.18^a^	1.11^a^	0.92^cd^	12.77^a^	4.59^ab^
6*	Special Yeast	M	N/A	0.49^b^	4.10^h^	13.59^defg^	79.18^fg^	0.21^f^	0.06^c^	0.05^fg^	9.80^abc^	3.95^abc^
7*	Dealcoholised	M	N/A	0.05^efg^	4.31^f^	25.34^a^	118.26^c^	0.09^f^	0.18^c^	0.09^fg^	3.85^cde^	2.22^abcde^
8	Dealcoholised	M	Hop extract	0.08^efg^	4.46^cd^	18.59^b^	153.61^b^	0.06^f^	0.18^c^	0.01^g^	0.00^e^	0.13^e^
9	Dealcoholised	M	Rice, Malt extract, Hop extract, Natural flavours	0.07^efg^	4.40^de^	5.26^I^	91.11^ef^	0.63^ef^	0.14^c^	0.24^fg^	2.90^de^	1.20^cde^
10	Dealcoholised	M	Sugar, Natural flavourings	0.08^efg^	4.13^gh^	11.34^gh^	71.25^g^	2.88^abc^	0.29^bc^	3.12^a^	0.00^e^	0.00^e^
11	Vacuum Distillation	M	Maize, Rice	0.03^fg^	4.10^h^	13.62^defg^	112.89^cd^	2.91^abc^	0.34^bc^	1.06^c^	4.61^bcde^	2.61^abcde^
12*	Vacuum Distillation	C	N/A	0.75^a^	4.27^f^	14.21^def^	235.98^a^	0.07^f^	0.23^c^	0.08^fg^	1.32^e^	1.65^bcde^
13*	Vacuum Distillation	C	N/A	0.35^cd^	4.19^g^	17.94^b^	98.22^def^	0.04^f^	0.07^c^	0.02^g^	0.21^e^	0.38^de^
14	Vacuum Distillation	M	Unmalted barley, Corn, Flavouring	0.39^c^	4.15^gh^	9.77^h^	154.62^b^	0.11^f^	0.19^c^	0.04^fg^	0.65^e^	1.36^cde^
15*	Mixed Methods	C	N/A	0.31^d^	4.38^e^	15.36^cde^	161.90^b^	2.18^abc^	0.25^c^	0.94^cd^	8.76^abcd^	3.28^abcd^
16*	Mixed Methods	M	Hop extract, Natural flavourings	0.12^e^	4.46^cd^	13.74^defg^	152.70^b^	0.90^def^	0.23^c^	0.41^efg^	3.78^cde^	1.41^cde^
17*	Mixed Methods	M	Corn	0.03^g^	3.99^I^	13.95^def^	109.88^cde^	2.05^abcd^	0.11^c^	2.00^b^	0.18^e^	0.17^e^
18	Mixed Methods	M	Maize, Natural flavourings	0.11^ef^	4.68^b^	12.94^efg^	92.30^ef^	1.90^bcde^	0.70^ab^	0.50^def^	10.69^ab^	3.44^abc^

### 2.2 Physicochemical analysis

Instrumental analyses were conducted to investigate the differences in the commercial non-alcoholic lager style beers and their key chemical characteristics. Ethanol content was measured using an Anton Paar Alcolyzer and DMA4500 (Graz, Austria). Sample pH was determined using a Metler Toledo FiveGo pH meter (Colombus, Ohio, USA) after calibration with pH 4.0 and 7.0 standards. Bitterness units (BU) were determined using the international method proposed by the American Society of Brewing Chemists (ASBC) (Beer-23A) (ASBC Method of Analysis, 2018). Beer (5 mL) was transferred into a 50 mL centrifuge tube and acidified with 3M HCl (0.5 mL). Isooctane (10 mL) was added and the mixture was shaken by hand three times and then placed on a mechanical shaker for 15 min. The mixture was subsequently centrifuged at 400 ×*g* for 5 min, and then again for another 5 min to aid phase separation. The clear isooctane layer was then transferred into a cuvette and absorbance was measured at 275 nm with a spectrophotometer against a blank of isooctane. The recorded absorbance was multiplied by 50 to give BU values in mg/L. Total polyphenol (TP) content was also determined using the international method proposed by the ASBC (Beer-35) (ASBC Method of Analysis, 2015). Beer (10 mL) was mixed with a preparation of carboxymethylcellulose (CMC, 1%) and ethylenediamine tetraacetic acid (EDTA, 0.2%) (8 mL) in a 25 mL volumetric flask. Ferric acid (0.5 mL) and ammonia (0.5 mL) were then added, with mixing after each addition. The solution was then made up to the mark with RO water, left to stand at room temperature for 10 min and absorbance was measured at 600 nm with a spectrophotometer against a blank of the beer sample (mixed with CMC/EDTA and ammonia). The recorded absorbance was multiplied by 820 to give total polyphenol values in mg/L. Fermentable sugars were determined via high-performance liquid chromatography (HPLC) using Dionex ICS-3000 Reagent-Free Ion Chromatography, electrochemical detection using ED40 and computer controller. The CarboPac PA20 column (3 x 150mm) was used, and the mobile phase was 10 mM NaOH with a flow rate of 0.5 mL/min. The injection volume was 10 μL and the column temperature was 30 °C. This method was modified from Kostas, White, Du, and Cook (2016). Authentic standards of sugars (maltose, sucrose, fructose, maltotriose, glucose) (Sigma-Aldrich Ltd, Dorset, UK) were used for quantification.

Gas Chromatography-Mass Spectrometry Flame Ionization Detector Headspace (GC–MS-FID-HS) lower boiling point beer volatile analysis was determined using the method proposed by Analytica-European Brewing Convention (EBC) (9.39) (Analytica-EBC, 2018). Beer samples (10 mL) were transferred into glass vials with 3.5 g sodium chloride and 50 µL 1-butanol (internal standard). Volatiles were analysed with a Scion 456-Gas Chromatograph (Scion Instruments, West Lothian, UK). Samples (500 µL) were incubated at 60 °C for 20 min with shaking, and then were injected in splitless mode using a PAL Combi-XT autosampler (PAL System, Zwingen, Switzerland) onto a Zebron ZBWax column (60 m × 0.25 ID; Phenomenex Inc, Cheshire, UK). Column temperature was held initially at 85 ˚C for 10 min, increased by 25 °C/min to 110 °C, before finally being increased by 8 °C/min to 200 °C. Total run time was 36.25 min. The GC carrier gas was helium, at a constant pressure of 15 psi. Full scan mode was used to detect volatile compounds (mass range from *m*/*z* 35 to 200). Volatile compounds were identified by their *m*/*z*, and quantified with the use of pure and internal standards. The following aroma compounds were purchased from Sigma-Aldrich (UK) for standard identification: acetaldehyde (≥99.5%), ethyl acetate (≥99.5%), isobutyl acetate (2-methylpropyl ethanonate) (≥97%), propan-1-ol (≥99%), isoamyl acetate (3-methylbutyl acetate) (≥97%), 3-methyl-1-butanol (≥99%), ethyl octanoate (≥98%) and ethyl decanoate (≥98%). Other compounds were purchased from Thermo Fisher Scientific (UK): 1-butanol (≥99.5%), ethyl butanoate (≥99%), 2-methylpropan-1-ol (≥99%) and ethyl hexanoate (≥99%).

To detect other relevant volatile compounds not found through GC–MS-FID-HS analysis, Solid Phase Microextraction (SPME) was used. Beer samples (5 mL) were transferred into glass vials and 100 µL 3-heptanone (internal standard) was added and analysed using a modified published method by Yang, Liu, Liu, Degn, Munchow, and Fisk (2016). Modifications to the method included incubation of samples at 40 °C for 2 min with shaking, with volatile aroma compounds extracted for 10 min and desorped for 1 min. Column temperature was held initially at 40 °C for 2 min, increased by 8 °C/min to 240 °C and held for 1 min. Total run time was 38 min. Full scan mode was used to detect volatile compounds (mass range from *m*/*z* 35 to 200). Volatiles were identified by their *m*/*z* and comparison of each mass spectrum with either the spectra from authentic compounds or with spectra in reference libraries (NIST/EPA/NIH Mass Spectral Library, version 2.0, Faircom Corporation, U.S.) The quantification of volatiles collected from the headspace was expressed by the peak area ratio (PAR), which was calculated by the GC peak area for the compound divided by the peak area of the internal standard.

### 2.3 Sensory analysis

Approvals from the University of Nottingham Medical Ethics Committee for both Quantitative Descriptive Analysis (QDA) and the Consumer Study (approval codes: 163–1812 and 328–1906) were granted. All participants gave written informed consent to participate and were offered an inconvenience allowance for their time. All tests took place at the Sensory Science Centre, Sutton Bonington Campus, University of Nottingham in individual booths conforming to ISO standards (ISO 8589: 2007). Data was collected using Compusense software (Guelph, Ontario, Canada).

#### 2.3.1 Quantitative descriptive analysis

The sensory attributes of eighteen commercial non-alcoholic lager style beer samples were evaluated by trained beer panellists (n = 10, 4 male, 6 female) using a modified QDA approach. Panellists were trained over twenty one, two-hour sessions. Initial training sessions identified and evaluated aroma, taste, flavour and mouthfeel attributes for all commercial beer samples using attribute generation. Subsequent training sessions expanded the attribute list, with definitions and reference standards for each attribute (data not shown here, see Appendix Table 1). Only attributes which the panel agreed on by consensus and that discriminated amongst samples were used. These attributes and definitions were developed in reference to published literature (Langstaff and Lewis, 1993, Meilgaard et al., 1979). All attributes were evaluated using a continuous unstructured line scale, with marks converted to a score of ten for data analysis purposes. Panellist performance was continually monitored for discrimination, consistency and repeatability using blind replicate samples and samples spiked with reference standards. Retraining was conducted where necessary. Final sample evaluation started once the panel demonstrated adequate repeatability and discrimination.

Samples were evaluated in nine 2 h sessions over two months, allowing for triplicate evaluations of each sample by each panellist. Beer samples, labelled with three-digit codes, were served at 4 ± 2 °C and presented in a balanced, blocked and randomised presentation order, with 2 min breaks between each sample. Panellists were provided with three bottles of each sample (3 × 20 mL) during assessment to ensure temperature was kept constant throughout assessment and beers were fresh. Panellists were instructed to use their first bottle for aroma, with subsequent bottles being used for flavour, taste and mouthfeel attributes. The order of attributes was agreed with panellists before final evaluation took place, starting with the attribute that was perceived first and ending with the last. A maximum of seven samples were evaluated per two-hour session to ensure no carryover or fatigue effects. Unsalted crackers (Rakusens, Leeds, UK), honeydew melon (Sainsburys, Milton Keynes, UK) and Evian mineral water (Danone, Paris, France) were provided for palate cleansing.

#### 2.3.2 Consumer liking analysis

Consumers (n = 104, 47 men, 57 women), who self-reported consumption of beer at least once a month participated in the study. A subset of the samples (n = 11) were selected after analysis of the QDA data to represent samples with a wide range of sensory characteristics produced by different production methods (shown in Table 1). All consumers participated in two evaluation sessions over two weeks. Both sessions collected overall liking (OL) data using a 9-pt hedonic scale ranging from ‘dislike extremely’ to ‘like extremely’, with consumers rating six samples per session. In each session, samples were presented monadically using a randomised balanced design according to a Williams Latin Square. To minimise fatigue and carryover, consumers were given a forced 1 min break between each sample, and were told to take at least 2 sips of water (Evian, Danone, France) and consume unsalted crackers (Rakusens, Leeds, UK) during this break to cleanse their palate.

### 2.4 Statistical analysis

All data analysis was conducted using XLSTAT (19.01, Addinsoft, New York, USA).

#### 2.4.1 Physicochemical analysis

Analysis of variance (ANOVA) followed by Tukey’s Honest Significant Difference (HSD) post hoc test were conducted at p < 0.05 for instrumental analysis. All analyses were conducted in duplicate across three sample bottles from the same batch, with an average mean calculated.

#### 2.4.2 Quantitative descriptive analysis

A two factor ANOVA (sample, panellist) with interaction and Tukey’s HSD post hoc test was performed on QDA sensory results. A cluster analysis on mean scores of all sensory attributes was performed using agglomerative hierarchical clustering, employing a dissimilarity matrix with Euclidean distance and Ward’s method in the agglomeration (Yang, Kraft, Shen, MacFie, & Ford, 2019).

#### 2.4.3 Correlation between physicochemical and QDA

Principle component analyses (PCA) were carried out on mean scores of physicochemical and sensory attributes to explore relationships. Both datatsets used averaged scores across samples and only included sensory attributes and physicochemical results which significantly discriminated amongst the samples, as assessed by ANOVA. Sensory attributes were selected as one input matrix, with physicochemical analysis as supplementary variables.

#### 2.4.4 Consumer liking analysis

To determine if differences existed amongst samples in terms of consumer overall liking a mixed model two-factor ANOVA (sample, consumer), with consumer as a random effect, was performed followed by Tukey’s HSD post hoc test. A cluster analysis on the overall liking data was also performed, to see if liking patterns varied across consumers, using agglomerative hierarchical clustering employing a dissimilarity matrix with Euclidean distance and Ward’s method in the agglomeration. A correlation test (Pearson’s correlation coefficient) between each individual’s result and cluster means was also performed to check the validity of cluster groups (Yang, Kraft, Shen, MacFie, & Ford, 2019). Differences amongst samples within each cluster was explored through further analyses with a two-factor ANOVA. An internal preference map with PCA biplot of multivariate space of non-alcoholic lagers was also configured, using average overall liking scores of consumer clusters and QDA sensory attributes as supplementary variables, to better visualize the data and understand drivers of like and dislike for each cluster.

## 3 Results and discussion

### 3.1 Physicochemical analysis of non-alcoholic beers

Instrumental analysis results for alcohol by volume (ABV), pH, bitterness units, total polyphenols and sugars can be found in Table 1. The ABV (%) of the NABs varied from 0.03 to 0.75 ABV. Although legal labelling criteria is different amongst countries, anything above 0.5% ABV cannot be classed as NAB (Department of Health & Social Care, 2018), therefore samples 2 and 12 cannot be described as NAB. It is interesting to note that both these beers were produced by craft breweries, posing the question whether the correct controls are in place to measure the final product ethanol concentration before bottling. Differences amongst the beers in terms of ABVs were explored due to the different production methods used. It has been well documented that membrane based dealcoholisation processes are not economically viable to produce a beer <0.5% ABV (Pilipovik & Riverol, 2005) and therefore it is suggested here that samples 7, 8, 9 and 10 were produced through other physical methods, which may include spinning cone column. The majority of samples produced by vacuum distillation (12, 13 and 14) were shown to have the highest ABVs (0.75, 0.35 and 0.39 respectively), apart from sample 11 which had one of the lowest ABVs (0.03). It is believed that this trend could again be due to the economic feasibility of this process (Müller, Bellut, Tippmann, & Becker, 2017). Overall it seemed that there was variation in each of the production methods in terms of ethanol content, but generally dealcoholised beers had the lowest ABV, whilst beers produced by vacuum distillation had the highest. All beers had values within the scope of previously obtained results for pH, BU and TP for commercial beers, ranging from 3.99 to 4.81 for pH, 5.26 to 25.34 for BU and 49.20 to 235.98 mg/L for TP (Briggs, Boulton, Brookes, & Stevens, 2004). Samples 7 and 8 had the highest concentration of BU, with both of these samples being produced by physical dealcoholisation, agreeing with similar studies that physical processes produced beers with higher BU (Schmelzle, Lindemann, & Methner, 2013). Sample 12 had the highest TP and this was the only sample that was unfiltered, so these polyphenols would not have been removed. The fermentable sugars measured were found to be higher in comparison to standard ABV beers (most notably for beers 1, 4, 5, and 8), which is proposed to be related to the production method used. Previous research has shown that biological production techniques produced beers with increased content of non-fermentable dextrins as the oligo- and polysaccharides in wort are not metabolized by yeast (Krebs, Müller, Becker, & Gastl, 2018). A clear differentiation in NABs produced by physical and biological methods due to differences in presence of sugars has been reported (Schmelzle, Lindemann, & Methner, 2013), yet here it is shown that there are now products on the market which do not follow this rule. For example, samples 10 and 11 (produced by dealcoholisation and vacuum distillation) had higher maltose, and sample 2 (produced by altered brewing) had smaller amounts of this sugar, revealing that other factors influenced the presence of sugars.

GC–MS-FID-HS analysis allowed identification of the most abundant compounds in beer, which included higher alcohols, esters and aldehydes (shown in Table 2). All compounds, except ethyl octanoate and ethyl decanoate, were significantly different amongst the eighteen samples (p < 0.05). The volatile compounds identified varied amongst samples, showing that NABs have a broad range of flavour characteristics. The presence or absence of these compounds was explored in relation to production methods. In terms of higher alcohols, sample 16 was found to have increased levels of 3-methyl-1-butanol (93.97 mg/L), followed by sample 4 (54.75 mg/L) compared to other beers. Samples 2 and 4 had increased amounts of 2-methylpropan-1-ol and propan-1-ol. Higher alcohols are the precursors to most flavour active esters, therefore when fermentation is halted prematurely in the brewing process these higher alcohols do not have sufficient time to be converted into esters (Briggs et al., 2004). Thus these samples were also found to have significantly reduced amounts of ethyl acetate and isoamyl acetate. Samples 7 and 8 (produced by dealcoholisation) were found to have none of these higher alcohols, agreeing with previous research that dealcoholisation removed a large amount of these important volatiles due to similarities with ethanol in terms of boiling point or molecular size (Müller, Bellut, Tippmann, & Becker, 2017). In terms of esters, samples 4, 9 and 16 (all produced using different production methods) had increased levels of ethyl acetate in comparison to other samples. Samples 9 and 16 also had increased amounts of isoamyl acetate. It is believed that these samples had higher levels of these esters due to either the addition of natural flavourings, or due to current advances in technologies. One example of this is the capturing of flavour concentrates from dealcoholized beer through pervaporation, which can then be blended back with the beer to increase the flavour profile to that of a standard beer (Branyik et al., 2012). Beers produced by altered brewing (1, 2 and 4), had significantly more ethyl acetate than those produced by physical methods (7, 8 and 10). Acetaldehyde is also a key volatile in beer, which is often discussed as an ‘off-flavour’ which arises from oxidation (Briggs, Boulton, Brookes, & Stevens, 2004). Samples 7 and 14 contained the highest amount of this volatile compound and it is believed that this was due to poor bottling technique, increasing oxygen levels and leading to contamination from spoilage microorganisms (Sohrabvandi, Mousavi, Razavi, Mortazavian, & Rezaei, 2010). Interestingly, it was thought that the beers produced by craft breweries may contain more of these ‘off-notes’ due to limited control in the bottling process, but all samples produced by craft breweries (2, 12, 13 and 15) had lower amounts. These physicochemical measurements suggest that there are many factors influencing the presence and quantity of flavour compounds of NABs, which not only include production method but also pre and post processing methods.

**Table 2:** Concentration of most abundant volatile compounds and flavour thresholds in beer measured by GC–MS-FID-HS for each sample. All flavour threshold values were stated based on literature from Morten C. Meilgaard (1982). Different letters within a column^abc^ represent a significant difference amongst samples in terms of volatile concentrations (Tukey’s HSD, p < 0.05). Samples which have concentrations of volatile compounds greater than threshold are shown in bold. Samples with an asterisk (*) were those selected for the subset for consumer overall liking sensory analysis.

Volatile compounds (ppm)		Acetaldehyde	Ethyl Acetate	Isobutyl Acetate (2-Methylpropyl Acetate)	Propan-1-ol	Ethyl Butanoate	2-Methylpropan-1-ol	Isoamyl Acetate	3-Methyl-1-Butanol	Ethyl Hexanoate	Ethyl Octanoate	Ethyl Decanoate
Sample Number	Production Method											
1*	Altered Brewing	2.16^cd^	5.29^f^	0.00^c^	0.00^e^	0.00^f^	0.11^g^	0.67^d^	8.36^h^	0.05^c^	0.03	0.00
2*	Altered Brewing	1.95^cd^	2.08^g^	0.00^c^	7.70^b^	0.00^f^	3.03^c^	0.12^f^	17.68^fg^	0.00^h^	0.02	0.00
3*	Altered Brewing	3.76^bcd^	0.00^I^	0.00^c^	0.00^e^	0.00^f^	0.00^g^	0.00^f^	0.00^j^	0.00^h^	0.00	0.01
4	Altered Brewing	8.76^ab^	14.07^c^	0.00^c^	8.94^a^	0.00^f^	7.02^a^	0.92^c^	54.75^b^	0.09^b^	0.02	0.01
5*	Special Yeast	1.90^cd^	0.00^i^	0.00^c^	0.00^e^	0.00^f^	0.00^g^	0.00^f^	0.00^j^	0.00^h^	0.00	0.02
6*	Special Yeast	9.16^ab^	1.20^gh^	0.00^c^	5.30^c^	0.00^f^	4.81^b^	0.07^f^	33.17^d^	0.00^h^	0.01	0.00
7*	Dealcoholised	**11.74**^a^	0.12^i^	0.00^c^	0.00^e^	0.00^f^	0.00^g^	0.00^f^	0.00^j^	0.00^h^	0.00	0.02
8	Dealcoholised	1.47^cd^	0.00^i^	0.00^c^	0.00^e^	0.00^f^	0.00^g^	0.00^f^	0.00^j^	0.00^h^	0.00	0.02
9	Dealcoholised	0.77^d^	**31.95**^a^	0.00^c^	0.00^e^	0.01^def^	0.00^g^	**4.24^b^**	42.55^c^	0.03^def^	0.00	0.01
10	Dealcoholised	1.59^cd^	0.20^i^	0.02^b^	0.00^e^	0.02^cd^	2.20^e^	0.43^e^	34.79^d^	0.05^cd^	0.02	0.03
11	Vacuum Distillation	4.05^bcd^	0.40^hi^	0.02^b^	0.00^e^	0.02^cd^	0.14^g^	0.99^c^	7.73^h^	0.02^efg^	0.00	0.02
12*	Vacuum Distillation	6.12^abcd^	5.24^f^	0.00^c^	2.97^d^	0.01^ef^	3.28^c^	0.39^e^	21.06^ef^	0.02^fgh^	0.01	0.00
13*	Vacuum Distillation	1.45^cd^	8.72^d^	0.08^b^	0.85^e^	0.05^a^	2.56^de^	0.68^d^	13.95^g^	0.02^fg^	0.03	0.00
14	Vacuum Distillation	**11.62**^a^	6.96^e^	0.17^a^	3.05^d^	0.02^cde^	2.94^cd^	0.61^d^	24.23^e^	0.04^cde^	0.02	0.01
15*	Mixed Methods	4.26^bcd^	1.56^g^	0.00^c^	3.46^d^	0.00^f^	1.38^f^	0.11^f^	6.77^hI^	0.01^gh^	000	0.00
16*	Mixed Methods	7.91^abc^	16.01^b^	0.00^c^	0.00^e^	0.02^cde^	0.00^g^	**6.18**^a^	**93.97**^a^	0.14^a^	0.02	0.02
17*	Mixed Methods	3.45^bcd^	1.41^g^	0.00^c^	0.00^e^	0.04^ab^	0.00^g^	0.33^e^	6.98^hI^	0.03^def^	0.00	0.00
18	Mixed Methods	1.55^cd^	0.02^i^	0.00^c^	0.25^e^	0.03^bc^	0.00^g^	0.34^e^	2.94^Ij^	0.03^cdef^	0.00	0.02
P Value		<0.0001	<0.0001	<0.0001	<0.0001	<0.0001	<0.0001	<0.0001	<0.0001	<0.0001	0.019	0.506
Flavour Threshold in Beer (ppm)		10	30	1.60	800	0.4	200	1.2	70	0.21	0.9	1.5

### 3.2 Descriptive sensory analysis of non-alcoholic beers

**Fig. 2:**
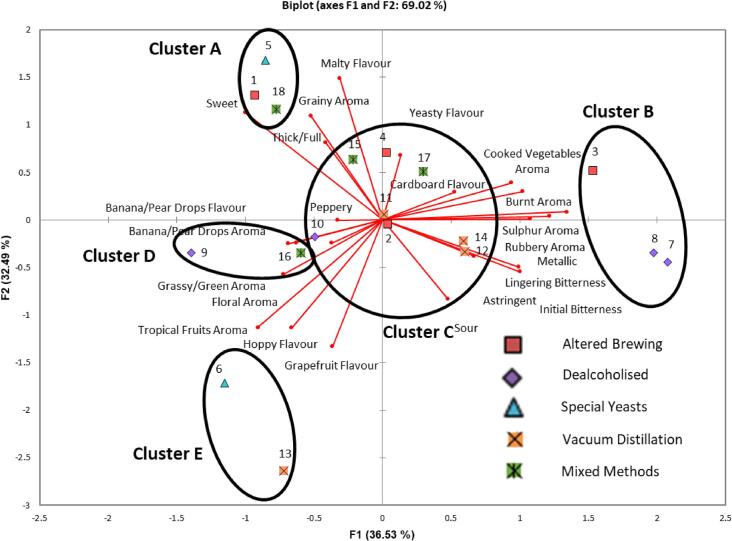
Principal component analysis (PCA) biplot of significant attributes present on principle component 1 and 2 by the covariance of mean significant attribute intensity ratings across non-alcoholic commercial lager samples with different production methods. Clusters of samples with similar sensory attributes, analysed using agglomerative hierarchical clustering, are circled and labelled in black.

Mean attribute scores and results from significance testing were calculated for all eighteen commercial NABs, using QDA with the trained panel (data not shown, Appendix Table 2). ANOVA revealed that for all twenty-three attributes, significant product differences were found (p < 0.0001). The data was clearly visualised by the use of a PCA (Fig. 2), showing the multivariate space of the NABs and their sensory attributes. The first two principal components (PCs) of the model accounted for 69.02% of variation in the data (36.53% and 32.49% for PC1 and PC2, respectively). PC1 was strongly positively correlated to cooked vegetable aroma (0.817), rubbery aroma (0.882), sulphur aroma (0.925), burnt aroma (0.890), initial (0.806) and lingering (0.806) bitterness, cardboard flavour (0.756), metallic (0.941) and astringent (0.790). PC1 was negatively correlated with floral aroma (−0.696) and sweet (−0.620). PC2 was strongly positively correlated with grainy aroma (0.756), thick/full (0.805), sweet (0.729), malty flavour (0.894) and yeasty flavour (0.822) and negatively correlated with tropical fruits aroma (−0.694), grapefruit flavour (−0.819), hoppy flavour (0–0.668) and sour (−0.805). PC3 (not shown) explained 15.88% of variance in the data and this is due to being strongly positively correlated with banana pear drops aroma (0.850) and flavour (0.879) and peppery (0.680) and negatively correlated with hoppy flavour (−0.549).

Mean attribute scores were also subjected to cluster analysis (Fig. 2, dendogram shown in Appendix – Fig. 1) to determine whether distinct subgroups of NABs could be identified and clusters explained by production method. Five clusters were easily identifiable. Cluster A (1, 5 and 18) contained samples which were positively correlated to grainy aroma, malty flavour, sweet and thick/full and all were produced by different methods. It should be highlighted that the term ‘malty flavour’ used here included worty characteristics. During panel training, panellists recognised many of the samples had a ‘worty’ characteristic, confirmed through the use of a wort sample as a reference, however the descriptor ’malty flavour’ was selected by the panel (see Appendix Table 1). Cluster B (3, 7 and 8) contained samples correlated to cooked vegetable, sulphur and rubbery aromas, initial and lingering bitterness, metallic and astringent aftertastes. Samples 7 and 8 were made using dealcoholisation techniques, with no additional adjuncts, however sample 3 was made using altered brewing techniques with the addition of the adjunct corn (Table 1). This may explain the strong correlation with the attribute ‘cooked vegetable aroma’ for this sample. It may also help to explain why it is clustered with samples made using physical processes as these methods have been previously associated with the above attributes (Schmelzle, Lindemann, & Methner, 2013). Cluster C, the largest cluster in this sample set (2, 4, 10, 11, 12, 14, 15 and 17) contained products not well described, receiving ratings close to the mean of the attributes, showing that these beers had a rather bland flavour profile. Cluster D contained samples (9 and 16) which were associated with banana pear drops aroma and flavour. These samples were found to contain ‘natural flavourings’, which may explain the banana/pear drop aroma and flavour characteristics. Peppery was an attribute that was discovered in sample 9 only, and this was in reference to the perception of heat/chilli. Previous research has looked at the effect of different irritants on their pungency using descriptive analysis (Cliff & Heymann, 1992) and found that ethanol brought burning and tingling sensations, with other irritants showing similar properties. It is therefore hypothesised that the commercial brewer for this sample could have introduced a similar irritant to counteract the lack of these sensations. However, common irritants such as eugenol, cinnamaldehyde and 4-vinylguaiacol (Cliff and Heymann, 1992, Lentz, 2018) were not found in GC–MS analysis. Cluster E (6 and 13) were found to have a hoppy aroma with high correlations to descriptors such as tropical fruits and floral aroma. It is believed that this was due to the samples being subjected to post-processing methods, such as dry hopping resulting in these aromas being perceived by the panel. Sample 13, was confirmed to be dry-hopped after the process.

It has previously been suggested that the production method used is the main factor for the differences in sensorial profiles of NAB (Schmelzle, Lindemann, & Methner, 2013), yet interestingly here this factor was not found to be the main driver of membership of beers within these clusters. Indeed, if this study had only categorised the samples into ‘biological and mixed methods’ and ‘physical’ production processes it would have shown a similar trend to that shown previously (Schmelzle, Lindemann, & Methner, 2013), whereby biological methods produced malty, worty and sweet beers, and physical methods produced bitter, sour and sulphur-like beers. However, here it appears that the sensory differences were due to other factors, such as pre and post processing methods, which reflects the increased development in this sector resulting in NABs with more complex sensorial profiles.

### 3.3 Correlation between physicochemical and descriptive sensory analysis results

Combining physicochemical and sensory results provides a comprehensive characterization of NABs. The correlation circle (as shown in Fig. 3a) shows sensory attributes with physicochemical results overlaid as supplementary data (further information on SPME-GC–MS data can be found in the Appendix, Table 3). As expected, attributes such as banana/pear drops aroma and flavour were projected similarly to volatile compounds well known for these attributes in beer; isoamyl acetate and 3-methyl-1-butanol. Fermentable sugars were also projected similarly with sensory attributes such as malty flavour, sweet and thick/full (Bellut & Arendt, 2019). An interaction between sweet and thick/full attributes in the QDA analysis revealed that all samples rated higher in terms of sweetness were also rated higher for thick/full. Total polyphenol content and bitterness units were correlated to initial and lingering bitterness, as well as astringency, with previous literature suggesting these physicochemical aspects relate to their sensory properties (Oladokun, Tarrega, James, Smart, Hort, & Cook, 2016). Interestingly, no compounds were identified to correlate to the attributes of cooked vegetable, burnt, sulphur and rubbery aroma, cardboard flavour or metallic. However, this may be due to the presence of highly odour active compounds at very low concentrations, such as sulfur compounds (dimethyl sulfide (DMS), dimethyl disulfide, dimethyl trisulfide, sulfur dioxide) which were not identified in the GC–MS analysis because the method was not sensitive or selective enough to identify them. Further work utilising a flame photometric detector (FPD) (Mundy, 1991) or sulfur chemiluminescence detection (SCD) (Burmeister et al., 1992) is therefore suggested to understand the presence of sulfur compounds in NABs and their contribution to these attributes.

**Fig. 3a:**
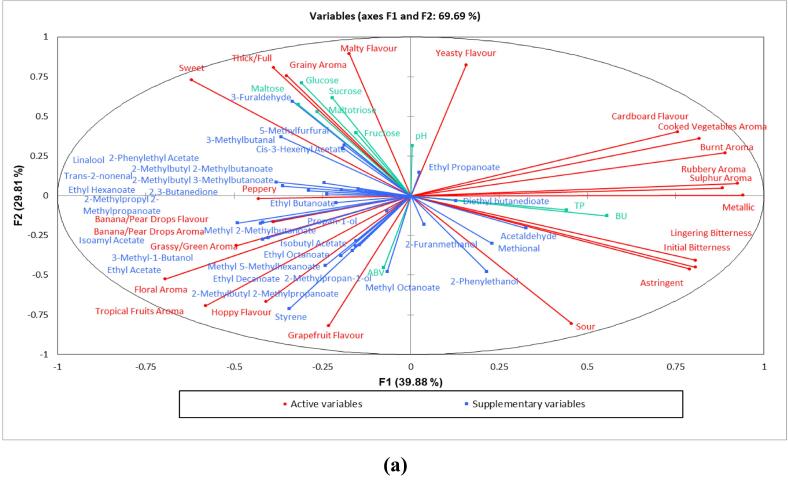
Correlation biplot of all physicochemical, instrumental and sensory data showing significant attributes present on principle component 1 and 2. Attributes in red show QDA sensory attributes, those in green show instrumental analysis and those in blue show volatile compounds found through GC–MS. (For interpretation of the references to colour in this figure legend, the reader is referred to the web version of this article.)

Overall the 18 samples were found in different locations of the PCA plot (as shown in Fig. 3b), reflecting the distinctive physicochemical and sensorial properties amongst the samples. PC1 was not correlated with any of the physicochemical data, yet samples 3, 7 and 8 (cluster B) were all positively correlated with this PC. Samples 7 and 8, had cooked vegetable aroma, sour and bitter tastes, with previous studies finding similar results and correlating this to the presence of DMS (Müller, Bellut, Tippmann, & Becker, 2017). Interestingly, it has been discussed that these ‘off-flavours’, as well as bitterness, become more dominant if other volatile compounds are removed to below threshold level, meaning the synergistic effects of the overall beer flavour become unbalanced (Gernat et al., 2019, Müller et al., 2017). This appears to be the case for these two samples, as they were only found to contain acetaldehyde in the lower boiling point volatile analysis. In addition to this, these samples were found to have decreased thick/full sensory ratings.

**Fig. 3b:**
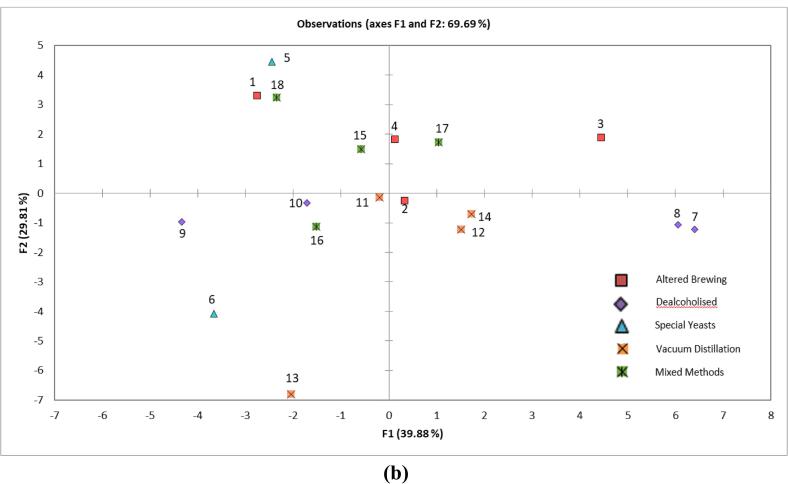
Principal component analysis (PCA) biplot of samples present on principle component 1 and 2 by the covariance of mean significant attribute intensity ratings by QDA and mean of instrumental analysis across non-alcoholic commercial lager samples with different production methods.

PC2 was strongly correlated with glucose (0.710), sucrose (0.614), maltose (0.575), maltotriose (0.529) and furfural (0.594). Samples 1, 5 and 18 (Cluster A) were situated close together and were positively correlated with PC2, with a grainy aroma, malty flavour, sweet and thick/full. These samples had increased levels of fermentable sugars, as well as 3-methylbutanal and furfural. Previous literature found that many factors can enhance the perception of undesirable sensory characteristics of ‘worty’ and ‘potato-like’ in beers, including; presence of significant amounts of aldehydes (furfural, 2-methylbutanal, 3-methylbutanal and 3-methylthiopropionaldehyde) (Perpete & Collin, 2000), absence of higher alcohols and esters which have been found to help mask these off-flavours (Saison, De Schutter, Uyttenhove, Delvaux, & Delvaux, 2009) and the presence of increased amounts of fermentable sugars (Perpete & Collin, 2000).

PC2 was negatively correlated with styrene (−0.713). Samples 6 and 13 were strongly correlated with this PC (Cluster E). They both had a sensorial profile of tropical fruits and floral aroma, grapefruit and hoppy flavour and sourness, which is likely to be due to the dry hopping technique employed for sample 13, and proposed here for sample 6 (although unconfirmed). Previous research has looked at increasing aroma intensity of low alcohol beer (1.2–1.4% ABV) by late hopping, and showed similar results to the current study of more intense fruit, citrus-like, green-grassy, and hop-spicy odour notes (Forster & Gahr, 2011) whilst also disguising off-flavours (such as styrene) by masking effects to improve overall aroma impression (Müller, Bellut, Tippmann, & Becker, 2017).

PC3 (data not shown here, see Appendix Fig. 2) was strongly correlated with ethyl acetate (0.529) and isoamyl acetate (0.687) and negatively correlated with 3-methylbutanal (−0.526) and methyl 2-methylbutanoate (−0.625). Samples 9, 10 and 16 were correlated with this PC (Cluster D). Sample 9 contained ethyl acetate and isoamyl acetate above threshold (31.94 mg/L and 4.24 mg/L respectively), and sample 16 also contained isoamyl acetate above threshold.

Finally, samples 2, 4, 11, 12, 14, 15 and 17 (Cluster C) were found to all be close to the centre of the PCA biplot, with similar means for all attributes. Samples 11, 12 and 14 in particular appear to be lacking volatiles, which agrees with the lack of specific sensory characteristics defining them.

Whilst the physicochemial and sensory data showed that resulting profiles did not appear to be related to production method when explored separately, when looking at this data together, some broad learnings appear. When comparing dealcoholized beers to those produced using biological methods, biological methods were found to have increased body. It is believed that this is due to brewers using a stepped mash profile, which consists of altering temperatures and timings to improve the body and mouthfeel of NABs (Branyik et al., 2012). Conversely, samples with decreased thick/full were found to follow previous literature that states that beers produced using physical methods have less body (Montanari, Marconi, Mayer, & Fantozzi, 2009). Samples 1 and 5 were both produced by biological production methods, with 5 being one of only two samples produced by special yeasts, and showing similar profiles of beer produced via this method to previous literature (Bellut & Arendt, 2019). Although the particular yeast strain used in this beer cannot be confirmed, previous research has suggested that *Saccharomyces ludwigii* is the most successful commercially available low alcohol yeast, used for industrial production (Branyik et al., 2012). It appears that all samples produced using vacuum distillation (12, 13, 14) were lacking in volatiles and dominant sensory attributes. Therefore, it seemed that this method removed a significant amount of volatiles, supporting previous literature which showed 76–97% of esters and 88–95% higher alcohols can be removed, due to similar boiling points to ethanol (Montanari, Marconi, Mayer, & Fantozzi, 2009). Interestingly, samples produced by this method had increased levels of 2-furanmethanol, which is a compound that serves as a marker for the heat load impact on the beer; in this case showing a small, but indeed relevant, heat-induced off-flavour (Gernat, Brouwer, & Ottens, 2019).

On the other hand, there are some samples which clearly did not follow a trend in relation to their production method. Samples 7, 8, 9 and 10 were all dealcoholised beers and whilst samples 7 and 8 followed previous literature with regards to their sensory properties, samples 9 and 10 showed completely different profiles. Samples 9 and 10 were shown to have ‘natural flavourings’ added to the ingredients list, suggesting this to be the cause. Interestingly, sample 3 (produced through interrupted fermentation) gave a similar sensory and physicochemical profile to samples 7 and 8 (produced by dealcoholisation methods). There is no clear explanation as to why this was the case, however it could be due to lack of vigour in the fermentation vessel during production, meaning that other compounds such as esters were not able to develop to mask these ‘off flavours’ (Saison, De Schutter, Uyttenhove, Delvaux, & Delvaux, 2009).

Therefore, the current data shows that the variation in sensory and physicochemical profiles of NABs may not only be due to the production methods used but also by other important factors including different starting raw materials (such as the addition of adjuncts including rye, wheat, rice or maize) or post processing methods (such as the use of additive flavour compounds, dry hopping or addition of liquid hop products post fermentation). One limitation of this study was that these beers were commercially produced, therefore it is difficult to draw conclusions on the real impact of production and pre and post processing methods on the sensory characteristics of these beers. It does however show, that there are a wide range of NABs with different sensory profiles on the current market, and the flavour profile of different production methods can be varied utilising different raw materials or post processing methods.

### 3.4 Consumer liking analysis

One of the key interests for the brewing industry is to understand the consumers most desired flavour profile for a NAB. This was explored through the use of a consumer panel registering their overall liking of a subset (n = 11) of the eighteen samples, selected from QDA for their range of flavour characteristics. In the initial analysis of overall liking for the eleven selected samples, significant differences were found (F (10, 1143) = 6.874, p = <0.0001), with samples 15 and 17 the most liked (mean = 6.221, SD = 1.393 and 1.966 respectively). These samples were both found to be in Cluster C, which were previously discussed to be perceived as having a bland flavour profile, with none of the sensory attributes rated highly for these samples. These beers were produced via mixed methods, indicating that overall liking for consumers could be optimised by mixing different production techniques. The samples that were least liked were samples 2 (mean = 5.058, SD = 2.189) and 7 (mean = 4.740, SD = 1.900), which were found to have significantly higher initial and lingering bitterness, as well as astringency. Subsequent application of agglomerative hierarchical clustering (AHC) analysis was performed to identify different clusters of consumers within the data set.

**Fig. 4:**
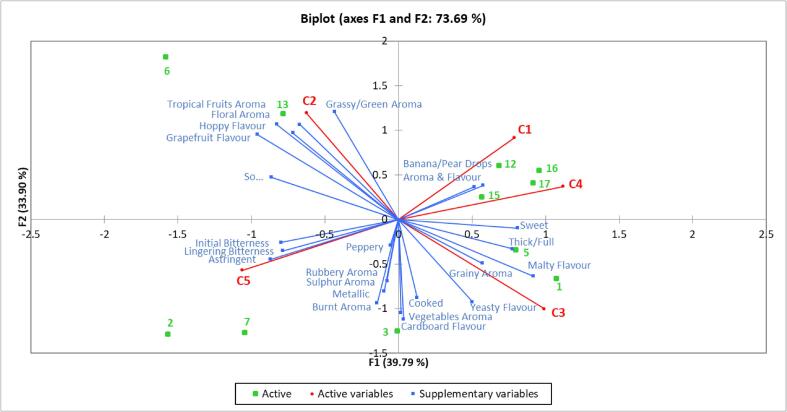
Internal preference map of mean overall liking data per cluster, with QDA sensory attributes as supplementary data. Red shows cluster number, green shows sample number and blue shows QDA sensory attributes.

Fig. 4 shows the internal preference map of the five consumer clusters identified. The ANOVA yielded significant differences for the interaction between sample and cluster (F (4, 1143) = 7.901, p = <0.0001), indicating that the overall liking of the samples varied with each consumer cluster. The first two principal components (PCs) of the model accounted for 73.69% of variation in the data (39.79% and 33.90% for PC1 and PC2, respectively). PC1 was strongly positively correlated to C1 (0.531), C3 (0.668), C4 (0.756), thick/full (0.523), sweet (0.546) and malty flavour (0.618). PC1 was negatively correlated to C5 (−0.716), initial (−0.538) and lingering (−0.530) bitterness, grapefruit flavour (−0.647), hoppy flavour (−0.558), sour (−0.582) and astringent (−0.588). PC2 was strongly positively correlated with C1 (0.617), *C*2 (0.804), grassy/green aroma (0.813), tropical fruits aroma (0.717) and floral aroma (0.654), grapefruit flavour (0.642) and hoppy flavour (0.719). PC2 was negatively correlated with C3 (−0.677), burnt aroma (−0.708), cardboard flavour (−0.757) and yeasty flavour (−0.623) and metallic (−0.633). Statistically, scores for cluster 1 (C1, n = 28) showed differences for consumer liking (F (10, 307) = 10.027, p = <0.0001) with Tukey’s HSD test indicating the overall liking was lowest for samples 2 and 7 (p = <0.0001). These consumers were described as ‘bitter dislikers’, as they were positively correlated to PC1. This was negatively correlated with attributes initial and lingering bitterness and astringent, with these consumers disliking samples which were rated highest for these attributes. Cluster 2 (C2, n = 28) yielded differences amongst samples (F (10, 307) = 16.073, p = <0.0001) and showed consumers within this cluster liked samples 6 and 13 and disliked samples 1 and 5. These consumers were described as ‘hoppy likers’, as this cluster was positively correlated with PC2, which was in turn positively correlated to hoppy and grapefruit flavours. The samples they most liked were those that had been dry hopped and were also described as hoppy by the QDA panel. C3 (C3, n = 12) were found to like samples (F (10, 131) = 6.985, p = <0.0001) 1, 3, 16 and 17, and dislike samples 6 and 13, showing the opposite of C2. This was confirmed by a negative correlation to PC2 and therefore these consumers were described as ‘hoppy dislikers’. In a study of Brazilian beer consumers, it was found that the least preferred beer style in the sample set was India Pale Ale and this was linked to the samples being hop-forward with increased bitterness, as well as having a characteristic floral note (Jardim, de Souza, Machado, Pinto, Ramos, & Garavaglia, 2018), which was also found with samples 6 and 13 here. This could therefore explain why the consumers in this cluster did not like these samples. C4 (C4, n = 17) liked samples 15, 12, 17, 1 and 13 the most (F (10, 186) = 9.537, p = <0.0001) and sample 2 the least. This cluster was positively correlated to PC1, which was positively correlated with thick/full, sweet and malty flavour and thus these were described as ‘malty/sweet likers’. Previous research (Porretta & Donadini, 2008) has shown that overall preference is highest for a full bodied beer with a malty and sweet taste, and consumers within this cluster seemed to follow this trend. C5 (C5, n = 19) showed no difference in overall liking amongst the samples (F (10, 208) = 0.872, p = 0.560) and rated all samples as ‘like slightly’. Although this cluster was negatively correlated with PC1 and thus correlated with bitterness and astringency, consumers within this cluster showed no clear preference for any of the samples. Therefore they were described as ‘enthusiasts’, as their overall liking for all samples was higher than other clusters; a similar group was found in beers with different ethanol concentrations (Ramsey et al., 2018) and bread (Gellynck, Kühne, Van Bockstaele, Van de Walle, & Dewettinck, 2009).

The present study showed that there are key differences within a population for NAB liking, confirmed due to the large number of clusters, which has also been found for standard beers (Guinard, Uotani, & Schlich, 2001) and is a key finding for the brewing industry. When data was analysed at surface level, the most liked samples were those with a fairly bland flavour profile. Yet when clustering was applied, it became apparent that samples with strong flavour profiles are either enthusiastically liked or disliked, shown by clusters of ‘hoppy likers’ and ‘hoppy dislikers’. This suggests that in the NAB sector, no one size fits all and therefore a company could be missing key insights by only looking at the mean data. Furthermore, this data shows that a variety of NABs with different sensory profiles are required to satisfy different consumer groups.

Finally, the overall liking score range amongst all clusters was found to be narrow, with consumers citing that they ‘slightly liked’ or ‘slightly disliked’ samples, and this was similar to ranges found by Ramsey et al. (2018) in terms of 0% beer. Therefore this shows that improvements are still required in this product space to ensure consumers are provided with sensorially acceptable products. On the other hand, consumers did not strongly oppose any of the beers, so good progress in the sensory quality of NABs is being made. It is important to note that the number of consumers per cluster were too low to draw strong conclusions so results for each cluster can only be viewed as trends in consumer data. Suggestions for future work are therefore to replicate the study with a larger group of consumers to understand the robustness of consumer cluster trends. These results could be used to advance the understanding of consumer liking of NAB.

Overall, this study provides a greater understanding into the differences between commercial NAB using physicochemical and sensorial techniques, and highlights that pre and post production methods should be taken into consideration when exploring relationships with production method. Advancements in new technologies have seen increased product development in this sector, with this research providing insight into the consumer demand for a wide range of sensory characteristics for NABs.

## 4 Conclusion

This study used instrumental techniques, a trained sensory panel and a consumer panel to evaluate the differences in commercial NAB in terms of physicochemical properties, perception of sensory attributes and their influence on consumer liking. Overall it showed that there is a clear range of non-alcoholic lagers currently on the market in the E.U., as breweries increased development to satisfy increased consumer demand. Advances and improvements in pre and post processing methods and production techniques were also shown. Contrary to previous findings revealing that production methods are the main factor in altering the physicochemical and sensory properties of NAB, this study showed many exceptions due to the use of mixed methods and pre and post-production practices. It therefore poses the question whether pre-processing factors (such as raw materials used) or post-brewing processes (such as the use of additive flavour compounds or dry hopping) have more of an influence on the overall quality of NABs. These therefore may be utilised by breweries to produce a wide range of NABs with different sensory profiles that are liked by different consumer clusters. In terms of overall liking, five different clusters of consumers were found, showing different liking trends and therefore key differences within the population.

This research is important for the global brewing industry as it gives valuable insight regarding the sensory impact of pre and post processing methods on the development of new NABs. Brewers can use this as a guide to select their desired NAB sensory characteristics, helping to fill a void in their current repertoire. Altering the sensorial profile of NAB in this way could be valuable to smaller craft breweries who may not have the capabilities to purchase expensive dealcoholisation equipment.

## Declaration of Competing Interest

The authors declare that they have no known competing financial interests or personal relationships that could have appeared to influence the work reported in this paper.
