# Supplementary material for: Understanding the sensory and physicochemical differences between commercially produced non-alcoholic lagers, and their influence on consumer liking
Source: Food Chem X. 2021 Jan 8;9:100114. doi: 10.1016/j.fochx.2021.100114 (PMC7822955; doi:10.1016/j.fochx.2021.100114)
Supplement: Supplementary Data 1 [file mmc1.doc]

# Supplementary Material


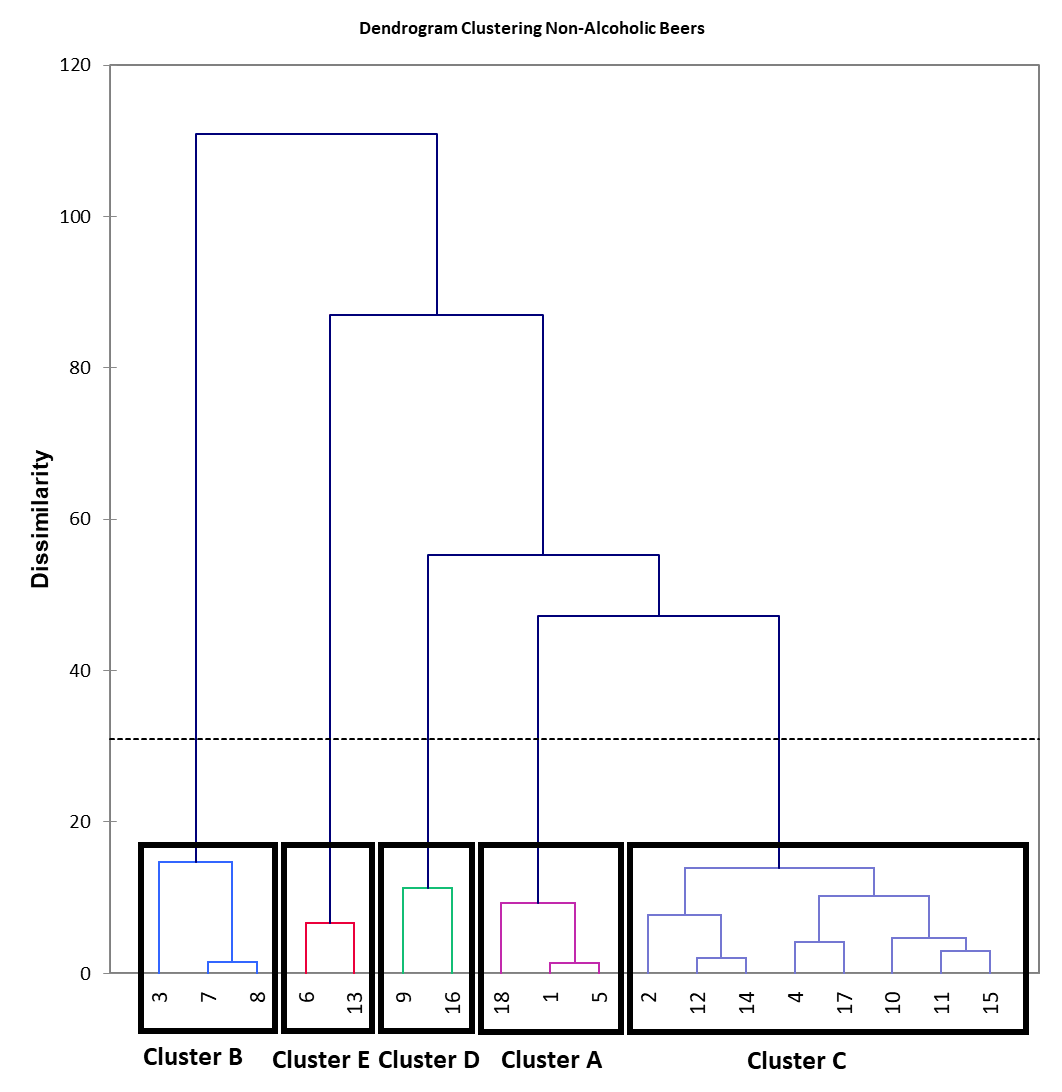


*Appendix Figure 1: Dendogram of agglomerative hierarchical clustering (AHC) of non-alcoholic beer samples*


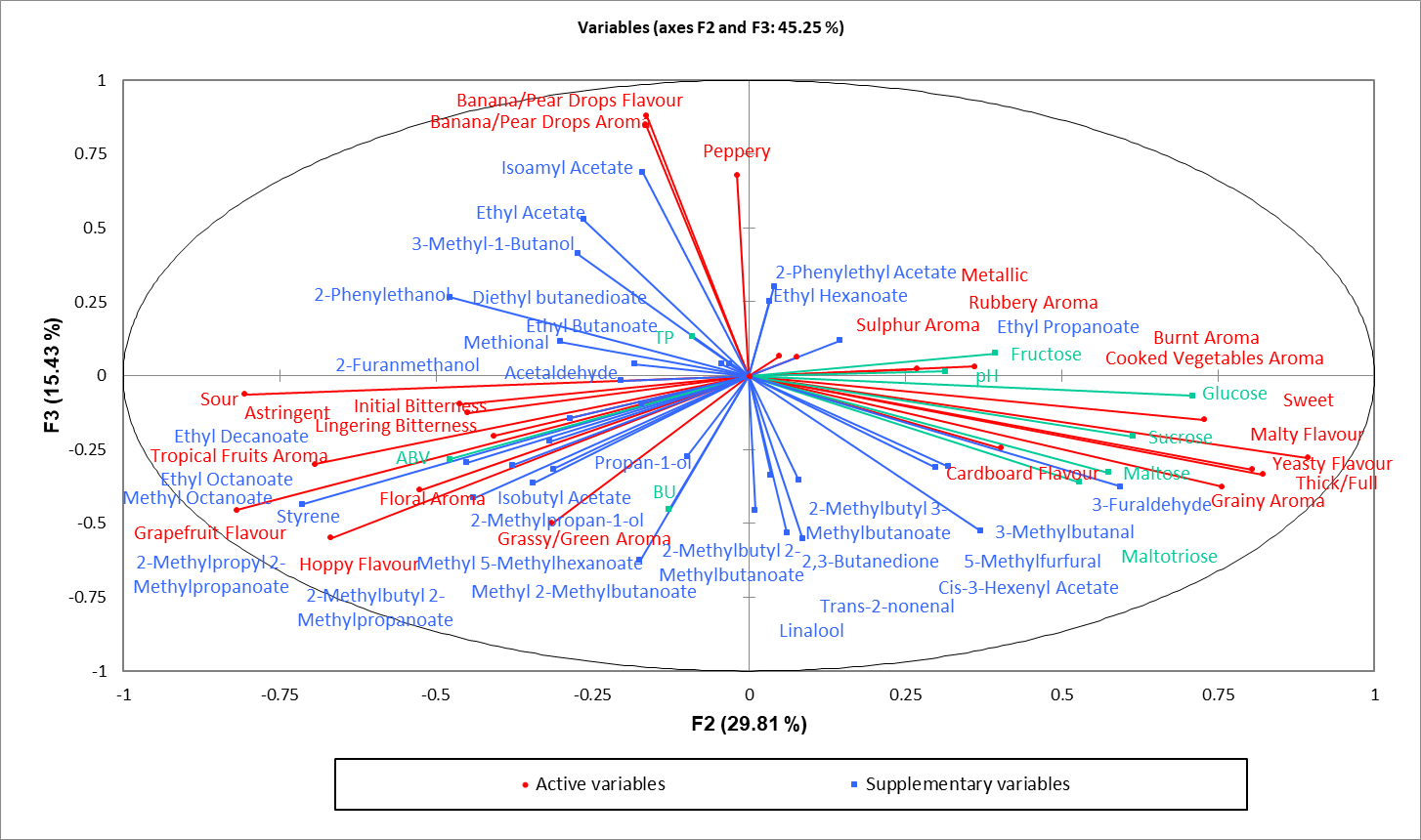


*Appendix Figure 2: Correlation biplot of all physiochemical, instrumental and sensory data showing significant attributes present on principle component 2 and 3. Attributes in red show QDA sensory attributes, those in green show instrumental analysis and those in blue show volatile compounds found through GC-MS*

*Appendix Table 1: Attributes, definitions and reference standards used in QDA trained sensory panel (n=10)*

|  | Attribute | Description | Reference |
| --- | --- | --- | --- |
| Aroma | Cooked Vegetables | Aroma associated with overcooked green vegetables such as cabbage, broccoli or Brussel sprouts or tinned sweetcorn (DMS) | 20ml water from overcooked boiled cabbage;  150ug DMS/L beer (AROXA™) |
|  | Rubbery | Aroma associated with rubber car tyres | N/A |
|  | Sulphur | Aroma associated with a struck match | 21mg sulphur dioxide/L beer (AROXA™) |
|  | Grassy/Green | Aroma associated with freshly cut grass or chopped leaves | 2.9mg cis-3-hexenol/L beer (AROXA™) |
|  | Banana/Pear Drops | Aroma associated with ripe or artificial banana and pear drops | 3.5mg isoamyl acetate/L beer (AROXA™) |
|  | Tropical Fruits | Overall intensity of aroma associated with tropical fruits including pineapple, mango, passionfruit and peach | 20ml tropical fruit juice |
|  | Floral | Aroma associated with flowers, particularly roses or violets | 4mg B-iodine/L beer (AROXA™); 1.2mg geraniol/L beer (AROXA™) |
|  | Grainy | Aroma associated with whole raw barley grain and hay/straw | 10g raw barley grain |
|  | Burnt | Aroma associated with burnt toast, dark roasted malt or burnt sugar (treacle) | 10g black treacle (Tate and Lyle) |
| Flavour | Banana/Pear Drops | Flavour associated with ripe or artificial banana and pear drops | 3.5mg isoamyl acetate/L beer (AROXA™) |
|  | Grapefruit | Flavour associated with freshly cut white grapefruit | 5g freshly cut white grapefruit flesh and skin |
|  | Hoppy | Flavour associated with fresh hops crushed in hand or hop pellets | 1.25mg hop oil extract/L beer (AROXA™) |
|  | Malty | Flavour associated with malt extract and fresh wort, which may also contain caramel notes | 50g malt extract mixed with 50ml water;  20ml fresh lager wort |
|  | Cardboard | Flavour associated with damp cardboard | 5g cardboard in 10ml water |
|  | Yeasty | Flavour associated with rehydrated yeast or bread dough | 5g bread yeast (Allinson) in 10ml water;  bread dough |
| Taste | Initial Bitterness | Taste stimulated by bitter substances such as caffeine or quinine | 13ul 30% iso-α-acids (TNS®) in 330ml water |
|  | Sweet | Taste stimulated by sucrose | 8.5mg sucralose/L beer (AROXA™) |
|  | Sour | Taste stimulated by acids | 457mg citric acid/L beer (AROXA™) |
|  | Lingering Bitterness | Persistence of bitterness in mouth, perceived 20 seconds after swallowing | 13ul 30% iso-α-acids (TNS®) in 330ml water |
| Mouthfeel | Thick/Full | Perception of thickness/fullness and syrupy mouthcoating, as beer is moved around in mouth. | N/A |
|  | Metallic | The taste of blood or iron, perceived 20 seconds after swallowing | 8.2mg ferrous sulphate/L beer (AROXA™) |
|  | Peppery | The perception of heat/chilli in back of throat and tip of tongue, perceived 30 seconds after swallowing | 20ml ginger beer (Old Jamaica) |
|  | Astringent | The feeling of drying/mouth puckering in mouth after swallow, perceived 30 seconds after swallowing | 1% tannic acid solution in water |

Appendix Table 2: Mean intensity of significant aroma, flavour, taste and mouthfeel attributes as evaluated by trained QDA panel. Different letters within a column represent a significant difference among samples based on differences in HSD (p<0.05)

|  | Aroma | | | | | | | | | | | | Flavour | | | | | | | | | | | Taste | | | | | | | Mouthfeel | | | | | |
| --- | --- | --- | --- | --- | --- | --- | --- | --- | --- | --- | --- | --- | --- | --- | --- | --- | --- | --- | --- | --- | --- | --- | --- | --- | --- | --- | --- | --- | --- | --- | --- | --- | --- | --- | --- | --- |
| Sample Name | Cooked Vegetables | | Rubbery | Sulphur | Grassy/Green | | Banana/Pear Drops | Tropical Fruits | Floral | | Grainy | Burnt | Banana/Pear Drops | | Grapefruit | Hoppy | | Malty | | Cardboard | Yeasty | | Initial Bitterness | | | Sweet | Sour | Lingering Bitterness | | Thick/Full | | Metallic | | Peppery | | Astringent |
| 1 | 1.01ᵉᶠᵍ | | 0.19ᵈ | 0.08ᵍ | 1.37ᵇᶜᵈᵉ | | 0.44ᵇᶜ | 1.38ᵇᶜᵈ | 1.69ᵇ | | 5.45ᵃᵇᶜ | 1.41ᵈᵉᶠ | 0.44ᵈ | | 0.62ᵇᶜ | 1.47ᶜ | | 7.65ᵃᵇ | | 1.94ᵃᵇᶜᵈᵉ | 2.75ᵃᵇᶜᵈ | | 3.15ᶠᵍ | | | 6.29ᵃᵇ | 1.29ᵉᶠ | 3.36ᵉᶠᵍʰ | | 6.11ᵃᵇ | | 2.02ᵈᵉᶠ | | 1.75ᵇ | | 3.96ᵉᶠᵍ |
| 2 | 1.34ᵈᵉᶠᵍ | | 0.10ᵈ | 1.02ᵈᵉᶠᵍ | 1.61ᵇᶜᵈᵉ | | 0.17ᵇᶜ | 1.01ᵇᶜᵈ | 1.48ᵇ | | 4.57ᵃᵇᶜᵈ | 0.97ᵈᵉᶠ | 0.09ᵈ | | 2.06ᵇ | 2.24ᵇᶜ | | 4.10ᵉᶠ | | 3.24ᵃ | 2.15ᵃᵇᶜᵈᵉ | | 5.18ᵇᶜᵈ | | | 2.51ᶠᵍʰ | 3.24ᵇᶜᵈ | 5.18ᵇᶜ | | 3.70ᵉᶠᵍ | | 3.55ᵇᶜᵈ | | 2.37ᵇ | | 5.96ᵃᵇᶜ |
| 3 | 4.91ᵃ | | 5.37ᵃ | 5.60ᵃ | 0.34ᵉ | | 0.08ᵇᶜ | 0.10ᵈ | 0.09ᵇ | | 1.81ᵉᶠ | 4.31ᵃᵇ | 0.53ᶜᵈ | | 0.94ᵇᶜ | 1.10ᶜ | | 4.47ᵈᵉᶠ | | 2.64ᵃᵇᶜ | 3.56ᵃ | | 4.62ᵇᶜᵈᵉᶠ | | | 2.65ᶠᵍʰ | 2.85ᵇᶜᵈᵉ | 4.70ᵇᶜᵈᵉ | | 4.12ᵈᵉᶠᵍ | | 4.93ᵃᵇ | | 1.19ᵇ | | 4.97ᶜᵈᵉᶠ |
| 4 | 1.83ᶜᵈᵉᶠᵍ | | 1.06ᶜᵈ | 1.55ᵈᵉᶠᵍ | 1.67ᵇᶜᵈᵉ | | 1.01ᵇᶜ | 0.93ᵇᶜᵈ | 1.27ᵇ | | 3.42ᶜᵈᵉ | 2.49ᵇᶜᵈ | 0.38ᵈ | | 0.79ᵇᶜ | 1.85ᵇᶜ | | 5.98ᵇᶜᵈ | | 2.09ᵃᵇᶜᵈᵉ | 3.10ᵃᵇᶜ | | 4.96ᵇᶜᵈᵉ | | | 5.07ᵇᶜᵈ | 2.10ᵈᵉᶠ | 4.77ᵇᶜᵈᵉ | | 5.37ᵃᵇᶜᵈ | | 4.63ᵃᵇᶜ | | 2.21ᵇ | | 5.03ᶜᵈᵉᶠ |
| 5 | 1.84ᶜᵈᵉᶠᵍ | | 0.37ᵈ | 0.60ᶠᵍ | 1.04ᵈᵉ | | 0.01ᶜ | 0.80ᶜᵈ | 1.20ᵇ | | 5.97ᵃᵇ | 1.52ᵈᵉᶠ | 0.10ᵈ | | 0.57ᶜ | 1.95ᵇᶜ | | 8.38ᵃ | | 2.00ᵃᵇᶜᵈᵉ | 3.18ᵃᵇᶜ | | 2.95ᵍ | | | 7.08ᵃ | 1.20ᵉᶠ | 2.89ᵍʰ | | 6.73ᵃ | | 2.60ᵈᵉᶠ | | 1.37ᵇ | | 3.45ᵍ |
| 6 | 0.70ᶠᵍ | | 0.36ᵈ | 0.26ᶠᵍ | 2.96ᵃᵇ | | 0.20ᵇᶜ | 5.39ᵃ | 4.25ᵃ | | 1.76ᵉᶠ | 0.38ᵉᶠ | 0.32ᵈ | | 6.00ᵃ | 7.20ᵃ | | 2.07ᵍʰ | | 1.01ᶜᵈᵉ | 1.09ᵈᵉ | | 4.15ᶜᵈᵉᶠᵍ | | | 3.22ᵉᶠᵍ | 3.76ᵃᵇᶜᵈ | 3.93ᶜᵈᵉᶠᵍ | | 4.03ᵈᵉᶠᵍ | | 1.11ᶠ | | 1.19ᵇ | | 4.29ᵈᵉᶠᵍ |
| 7 | 4.51ᵃᵇ | | 4.39ᵃᵇ | 3.90ᵃᵇᶜ | 0.56ᵉ | | 0.01ᶜ | 0.01ᵈ | 0.01ᵇ | | 1.21ᶠ | 4.53ᵃ | 0.14ᵈ | | 1.64ᵇᶜ | 1.25ᶜ | | 2.80ᶠᵍʰ | | 2.86ᵃᵇ | 1.89ᵃᵇᶜᵈᵉ | | 7.90ᵃ | | | 0.86ᶦ | 4.42ᵃᵇ | 7.65ᵃ | | 3.17ᶠᵍ | | 5.77ᵃ | | 0.91ᵇ | | 6.94ᵃ |
| 8 | 4.03ᵃᵇᶜ | | 4.03ᵃᵇ | 4.78ᵃᵇ | 1.02ᵈᵉ | | 0.29ᵇᶜ | 0.02ᵈ | 0.16ᵇ | | 1.74ᵉᶠ | 4.02ᵃᵇᶜ | 0.22ᵈ | | 1.10ᵇᶜ | 1.68ᵇᶜ | | 3.49ᶠᵍ | | 3.53ᵃ | 1.45ᶜᵈᵉ | | 8.07ᵃ | | | 0.86ᶦ | 3.77ᵃᵇᶜ | 7.42ᵃ | | 2.69ᵍ | | 5.75ᵃ | | 1.49ᵇ | | 6.71ᵃᵇ |
| 9 | 1.32ᵈᵉᶠᵍ | | 0.49ᵈ | 0.22ᶠᵍ | 1.19ᵇᶜᵈᵉ | | 7.11ᵃ | 2.21ᵇᶜ | 1.66ᵇ | | 1.96ᵉᶠ | 0.44ᵉᶠ | 7.28ᵃ | | 1.02ᵇᶜ | 1.75ᵇᶜ | | 2.63ᶠᵍʰ | | 0.66ᵉ | 0.60ᵉ | | 2.94ᵍ | | | 3.99ᵈᵉᶠ | 2.55ᶜᵈᵉᶠ | 2.09ʰ | | 3.53ᵉᶠᵍ | | 1.34ᵉᶠ | | 4.89ᵃ | | 3.43ᵍ |
| 10 | 1.34ᵈᵉᶠᵍ | | 1.54ᶜᵈ | 0.88ᵉᶠᵍ | 1.21ᵇᶜᵈᵉ | | 1.40ᵇ | 2.49ᵇ | 1.71ᵇ | | 1.57ᵉᶠ | 0.53ᵉᶠ | 1.68ᶜ | | 1.10ᵇᶜ | 2.00ᵇᶜ | | 2.79ᶠᵍʰ | | 1.27ᵇᶜᵈᵉ | 2.04ᵃᵇᶜᵈᵉ | | 3.53ᵉᶠᵍ | | | 3.98ᵈᵉᶠ | 2.20ᶜᵈᵉᶠ | 3.15ᶠᵍʰ | | 3.70ᵉᶠᵍ | | 2.38ᵈᵉᶠ | | 1.65ᵇ | | 4.41ᵈᵉᶠᵍ |
| 11 | 2.27ᵇᶜᵈᵉᶠᵍ | | 1.32ᶜᵈ | 1.20ᵈᵉᶠᵍ | 1.57ᵇᶜᵈᵉ | | 0.88ᵇᶜ | 0.51ᵈ | 1.77ᵇ | | 2.69ᵈᵉᶠ | 0.99ᵈᵉᶠ | 1.71ᶜ | | 0.76ᵇᶜ | 1.43ᶜ | | 3.69ᵉᶠᵍ | | 1.89ᵃᵇᶜᵈᵉ | 1.52ᶜᵈᵉ | | 4.55ᵇᶜᵈᵉᶠ | | | 2.88ᶠᵍʰ | 2.53ᶜᵈᵉᶠ | 3.70ᵈᵉᶠᵍ | | 3.09ᶠᵍ | | 3.30ᵇᶜᵈᵉ | | 1.13ᵇ | | 4.70ᶜᵈᵉᶠ |
| 12 | 2.36ᵇᶜᵈᵉᶠᵍ | | 2.55ᵇᶜ | 2.06ᶜᵈᵉᶠ | 2.60ᵃᵇᶜᵈ | | 0.59ᵇᶜ | 0.53ᵈ | 0.34ᵇ | | 2.60ᵈᵉᶠ | 1.32ᵈᵉᶠ | 0.59ᶜᵈ | | 1.75ᵇᶜ | 1.98ᵇᶜ | | 3.56ᶠᵍ | | 2.49ᵃᵇᶜᵈ | 1.14ᵈᵉ | | 5.44ᵇᶜ | | | 1.46ʰᶦ | 4.44ᵃᵇ | 5.14ᵇᶜᵈ | | 3.26ᶠᵍ | | 3.57ᵇᶜᵈ | | 1.28ᵇ | | 5.03ᶜᵈᵉᶠ |
| 13 | 0.14ᵍ | | 0.34ᵈ | 0.18ᶠᵍ | 2.87ᵃᵇᶜ | | 0.65ᵇᶜ | 6.84ᵃ | 4.17ᵃ | | 1.06ᶠ | 0.07ᶠ | 0.68ᶜᵈ | | 7.16ᵃ | 7.61ᵃ | | 1.50ʰ | | 0.90ᵈᵉ | 0.46ᵉ | | 5.94ᵇ | | | 1.50ʰᶦ | 5.14ᵃ | 5.81ᵇ | | 2.57ᵍ | | 3.16ᵇᶜᵈᵉ | | 1.39ᵇ | | 5.76ᵃᵇᶜᵈ |
| 14 | 2.47ᵇᶜᵈᵉᶠ | | 1.98ᶜᵈ | 2.87ᵇᶜᵈ | 1.44ᵇᶜᵈᵉ | | 0.54ᵇᶜ | 0.71ᶜᵈ | 0.43ᵇ | | 2.19ᵉᶠ | 2.24ᶜᵈᵉ | 0.92ᶜᵈ | | 1.33ᵇᶜ | 2.12ᵇᶜ | | 3.39ᶠᵍ | | 2.55ᵃᵇᶜ | 1.70ᵇᶜᵈᵉ | | 4.55ᵇᶜᵈᵉᶠ | | | 1.76ᵍʰᶦ | 4.51ᵃᵇ | 4.08ᶜᵈᵉᶠᵍ | | 3.28ᶠᵍ | | 3.58ᵇᶜᵈ | | 1.38ᵇ | | 5.38ᵇᶜᵈᵉ |
| 15 | 2.34ᵇᶜᵈᵉᶠᵍ | | 0.87ᶜᵈ | 0.70ᵉᶠᵍ | 1.32ᵇᶜᵈᵉ | | 0.40ᵇᶜ | 1.03ᵇᶜᵈ | 1.00ᵇ | | 4.29ᵇᶜᵈ | 1.46ᵈᵉᶠ | 0.50ᶜᵈ | | 0.72ᵇᶜ | 1.52ᶜ | | 5.56ᶜᵈᵉ | | 2.47ᵃᵇᶜᵈ | 1.66ᵇᶜᵈᵉ | | 3.87ᵈᵉᶠᵍ | | | 4.05ᵈᵉᶠ | 2.20ᶜᵈᵉᶠ | 4.09ᶜᵈᵉᶠᵍ | | 4.35ᶜᵈᵉᶠ | | 2.76ᶜᵈᵉᶠ | | 1.21ᵇ | | 4.40ᵈᵉᶠᵍ |
| 16 | 1.11ᵈᵉᶠᵍ | | 0.17ᵈ | 0.34ᶠᵍ | 1.59ᵇᶜᵈᵉ | | 6.12ᵃ | 1.49ᵇᶜᵈ | 0.96ᵇ | | 1.73ᵉᶠ | 1.08ᵈᵉᶠ | 5.02ᵇ | | 1.05ᵇᶜ | 1.47ᶜ | | 3.52ᶠᵍ | | 1.36ᵇᶜᵈᵉ | 0.99ᵈᵉ | | 4.40ᶜᵈᵉᶠᵍ | | | 2.77ᶠᵍʰ | 2.52ᶜᵈᵉᶠ | 4.36ᶜᵈᵉᶠ | | 3.54ᵉᶠᵍ | | 3.01ᵇᶜᵈᵉᶠ | | 1.96ᵇ | | 5.03ᶜᵈᵉᶠ |
| 17 | 3.17ᵃᵇᶜᵈᵉ | | 1.87ᶜᵈ | 2.55ᶜᵈᵉ | 1.10ᶜᵈᵉ | | 0.64ᵇᶜ | 0.42ᵈ | 0.32ᵇ | | 2.91ᵈᵉᶠ | 1.77ᵈᵉᶠ | 1.14ᶜᵈ | | 1.14ᵇᶜ | 1.82ᵇᶜ | | 4.26ᵈᵉᶠ | | 2.75ᵃᵇ | 3.38ᵃᵇ | | 4.36ᶜᵈᵉᶠᵍ | | | 4.49ᶜᵈᵉ | 3.17ᵇᶜᵈ | 4.19ᶜᵈᵉᶠᵍ | | 4.87ᵇᶜᵈᵉ | | 3.75ᵇᶜᵈ | | 1.19ᵇ | | 4.48ᵈᵉᶠᵍ |
| 18 | 3.32ᵃᵇᶜᵈ | | 1.22ᶜᵈ | 0.63ᶠᵍ | 3.76ᵃ | | 0.18ᵇᶜ | 1.40ᵇᶜᵈ | 1.80ᵇ | | 6.36ᵃ | 1.34ᵈᵉᶠ | 0.25ᵈ | | 1.05ᵇᶜ | 3.49ᵇ | | 7.11ᵃᵇᶜ | | 2.64ᵃᵇᶜ | 3.54ᵃ | | 3.61ᵉᶠᵍ | | | 5.94ᵃᵇᶜ | 1.12ᶠ | 2.99ᶠᵍʰ | | 5.86ᵃᵇᶜ | | 1.99ᵈᵉᶠ | | 1.57ᵇ | | 3.86ᶠᵍ |
|  | |  | | | |  | |  | |  | |  | |  | | |  | |  | | |  | | |  | | | |  | | | |  | |  | |

*Appendix Table 3: Peak area ratio of compounds detected by SPME-GC-MS, with corresponding retention times. Different letters within a column****ᵃ****ᵇᶜ represent a significant difference among samples in terms of volatile concentrations (Tukey’s HSD, p<0.05).*

| Peak Number | 1 | 2 | 3 | 4 | 5 | 6 | 7 | 8 | 9 | 10 | 11 | 12 | 13 | 14 | 15 | 16 | 17 | 18 | 19 | 20 |
| --- | --- | --- | --- | --- | --- | --- | --- | --- | --- | --- | --- | --- | --- | --- | --- | --- | --- | --- | --- | --- |
| Name | 3-Methylbutanal | Ethyl Propanoate | Methyl 2-Methylbutanoate | 2-Methylpropyl 2-Methylpropanoate | 2-Methylbutyl 2-Methylpropanoate | Methyl 5-Methylhexanoate | Styrene | 2-Methylbutyl 2-Methylbutanoate | 2-Methylbutyl 3-Methylbutanoate | cis-3-Hexenyl Acetate | Methyl Octanoate | 3-Furaldehyde | Linalool | Trans-2-Nonenal | 5-Methylfurfural | 2-Furanmethanol | Diethyl butanedioate | Methional | 2-Phenylethyl Acetate | 2-Phenylethanol |
| Retention Time | 4.66 | 5.35 | 6.37 | 6.52 | 7.80 | 11.25 | 11.84 | 12.01 | 12.30 | 12.83 | 14.22 | 15.93 | 16.98 | 17.24 | 17.90 | 19.07 | 19.30 | 20.11 | 21.80 | 23.20 |
| Peak Area Ratio | 0.03ᵇᶜ | 0.00ᶠ | 0.04ᵃᵇᶜᵈ | 0.00ᵃᵇ | 0.08ᵈ | 0.00ᶠ | 0.23ᵇ | 0.00ᵇ | 0.01ᶜ | 0.00ᵇ | 0.01ᶜ | 0.02ᵈᵉ | 0.08ᵈ | 0.01ᵇ | 0.00ᵇᶜ | 0.01ᵍʰᶦʲ | 0.01ᵇ | 0.00ᶜ | 0.34ᶜ | 0.23ᶦʲᵏ |
|  | 0.01ᶜ | 0.00ᶠ | 0.04ᵃᵇᶜᵈ | 0.00ᵃ | 0.13ᵈ | 0.00ᶠ | 0.04ᵇ | 0.00ᵇ | 0.01ᶜ | 0.00ᵇ | 0.10ᵇᶜ | 0.01ᵉ | 0.08ᵈ | 0.00ᵇᶜ | 0.00ᵇᶜ | 0.00ᵏ | 0.00ᵇ | 0.00ᶜ | 0.02ᶠ | 0.15ʲᵏ |
|  | 0.01ᶜ | 0.00ᶠ | 0.04ᵇᶜᵈ | 0.00ᵃᵇ | 0.00ᵈ | 0.00ᶠ | 0.00ᵇ | 0.00ᵇ | 0.00ᶜ | 0.00ᵇ | 0.01ᶜ | 0.01ᵉ | 0.00ᶦ | 0.00ᶜ | 0.00ᵇᶜ | 0.01ᵉᶠᵍʰᶦ | 0.00ᵇ | 0.00ᶜ | 0.00ᶠ | 0.56ʰᶦ |
|  | 0.02ᵇᶜ | 0.18ᵃ | 0.04ᵃᵇᶜᵈ | 0.00ᵃᵇ | 0.04ᵈ | 0.00ᶠ | 0.01ᵇ | 0.00ᵇ | 0.01ᶜ | 0.00ᵇ | 0.00ᶜ | 0.05ᵇᶜ | 0.02ᶠᵍʰᶦ | 0.00ᶜ | 0.00ᵇ | 0.03ᶜᵈ | 0.00ᵇ | 0.00ᶜ | 1.04ᵇ | 1.30ᶠᵍ |
|  | 0.05ᵃ | 0.00ᶠ | 0.05ᵃᵇ | 0.00ᵃᵇ | 0.17ᵈ | 0.00ᶠ | 0.01ᵇ | 0.00ᵇ | 0.01ᶜ | 0.00ᵇ | 0.00ᶜ | 0.07ᵃᵇ | 0.04ᵉ | 0.00ᶜ | 0.00ᵇᶜ | 0.02ᵉᶠᵍʰ | 0.00ᵇ | 0.00ᶜ | 0.00ᶠ | 0.02ᵏ |
|  | 0.02ᵇᶜ | 0.00ᶠ | 0.05ᵃ | 0.01ᵃ | 10.64ᵃ | 0.03ᵃ | 0.69ᵃᵇ | 0.00ᵇ | 0.09ᵇᶜ | 0.00ᵇ | 0.01ᶜ | 0.01ᵉ | 0.17ᶜ | 0.01ᵇ | 0.00ᵇᶜ | 0.02ᵉᶠᵍ | 0.00ᵇ | 0.00ᶜ | 0.03ᶠ | 0.53ʰᶦʲ |
|  | 0.01ᵇᶜ | 0.00ᶠ | 0.04ᵇᶜᵈ | 0.00ᵃᵇ | 0.00ᵈ | 0.00ᶠ | 0.05ᵇ | 0.00ᵇ | 0.00ᶜ | 0.00ᵇ | 0.01ᶜ | 0.01ᵉ | 0.00ʰᶦ | 0.00ᶜ | 0.00ᶜ | 0.01ᶦʲᵏ | 0.00ᵇ | 0.00ᶜ | 0.00ᶠ | 1.47ᶠ |
|  | 0.02ᵇᶜ | 0.00ᶠ | 0.04ᵃᵇᶜᵈ | 0.00ᵃᵇ | 0.00ᵈ | 0.00ᶠ | 0.07ᵇ | 0.00ᵇ | 0.00ᶜ | 0.00ᵇ | 0.32ᵃᵇᶜ | 0.01ᵉ | 0.00ᶦ | 0.00ᶜ | 0.00ᵇᶜ | 0.02ᵈᵉᶠ | 0.00ᵇ | 0.00ᵇ | 0.01ᶠ | 2.05ᵉ |
|  | 0.01ᶜ | 0.01ᵉᶠ | 0.04ᵃᵇᶜᵈ | 0.00ᵃᵇ | 0.11ᵈ | 0.00ᶠ | 0.00ᵇ | 0.00ᵇ | 0.04ᶜ | 0.00ᵇ | 0.03ᶜ | 0.02ᵉ | 0.01ᵍʰᶦ | 0.00ᶜ | 0.00ᵇᶜ | 0.01ʲᵏ | 0.00ᵇ | 0.00ᶜ | 0.01ᶠ | 1.36ᶠ |
|  | 0.02ᵇᶜ | 0.00ᶠ | 0.04ᵇᶜᵈ | 0.00ᵃᵇ | 0.09ᵈ | 0.01ᶜ | 0.39ᵃᵇ | 0.00ᵇ | 0.05ᶜ | 0.01ᵇ | 0.09ᶜ | 0.01ᵉ | 0.03ᵉᶠ | 0.00ᶜ | 0.00ᵇᶜ | 0.02ᵈᵉ | 0.00ᵇ | 0.00ᶜ | 0.19ᵈ | 1.37ᶠ |
|  | 0.01ᵇᶜ | 0.00ᵉᶠ | 0.04ᵃᵇᶜᵈ | 0.00ᵃᵇ | 0.02ᵈ | 0.00ᵈᵉ | 0.00ᵇ | 0.00ᵇ | 0.00ᶜ | 0.00ᵇ | 0.24ᵇᶜ | 0.02ᵉ | 0.01ᵍʰᶦ | 0.00ᶜ | 0.00ᵇᶜ | 0.01ᶠᵍʰᶦʲ | 0.00ᵇ | 0.00ᶜ | 0.29ᶜ | 2.26ᵈᵉ |
|  | 0.02ᵇᶜ | 0.01ᵉᶠ | 0.04ᵃᵇᶜᵈ | 0.01ᵃ | 8.76ᵇ | 0.03ᵇ | 0.01ᵇ | 0.01ᵃ | 0.23ᵇ | 0.00ᵇ | 0.25ᵇᶜ | 0.02ᵉ | 0.02ᶠᵍʰ | 0.00ᶜ | 0.00ᵇᶜ | 0.03ᵇᶜ | 0.00ᵇ | 0.01ᵃ | 0.11ᵉ | 2.50ᶜᵈ |
|  | 0.03ᵇ | 0.01ᵉ | 0.05ᵃᵇᶜ | 0.00ᵃᵇ | 3.03 | 0.00ᵉ | 1.11ᵃ | 0.00ᵇ | 0.09ᵇᶜ | 0.00ᵇ | 0.70ᵃ | 0.01ᵉ | 0.19ᵇ | 0.01ᵇ | 0.00ᵇᶜ | 0.02ᵈᵉᶠᵍ | 0.00ᵇ | 0.00ᵇᶜ | 0.05ᵉᶠ | 3.30ᵇ |
|  | 0.01ᵇᶜ | 0.04ᵈ | 0.04ᶜᵈ | 0.00ᵃᵇ | 0.01ᵈ | 0.00ᶠ | 0.01ᵇ | 0.00ᵇ | 0.00ᶜ | 0.00ᵇ | 0.03ᶜ | 0.02ᵉ | 0.00ʰᶦ | 0.00ᶜ | 0.00ᵇᶜ | 0.04ᵃ | 0.11ᵃ | 0.00ᵇᶜ | 0.18ᵈ | 5.04ᵃ |
|  | 0.02ᵇᶜ | 0.00ᶠ | 0.04ᵃᵇᶜᵈ | 0.00ᵃᵇ | 0.10ᵈ | 0.00ᶠ | 0.05ᵇ | 0.00ᵇ | 0.00ᶜ | 0.00ᵇ | 0.01ᶜ | 0.04ᶜᵈ | 0.02ᵉᶠᵍ | 0.00ᶜ | 0.00ᵇᶜ | 0.03ᵇᶜᵈ | 0.00ᵇ | 0.00ᵇᶜ | 0.04ᶠ | 0.90ᵍʰ |
|  | 0.02ᵇᶜ | 0.10ᶜ | 0.04ᵈ | 0.00ᵃ | 0.03ᵈ | 0.00ᶠ | 0.03ᵇ | 0.00ᵇ | 0.00ᶜ | 0.00ᵇ | 0.21ᵇᶜ | 0.01ᵉ | 0.00ᶦ | 0.00ᶜ | 0.00ᵇᶜ | 0.03ᵃᵇ | 0.00ᵇ | 0.00ᵇᶜ | 1.43ᵃ | 3.39ᵇ |
|  | 0.01ᶜ | 0.17ᵇ | 0.04ᵃᵇᶜᵈ | 0.00ᵇ | 0.02ᵈ | 0.00ᶠ | 0.02ᵇ | 0.00ᵇ | 0.00ᶜ | 0.00ᵇ | 0.02ᶜ | 0.01ᵉ | 0.02ᶠᵍʰᶦ | 0.00ᶜ | 0.00ᵇᶜ | 0.01ᶦʲᵏ | 0.00ᵇ | 0.00ᶜ | 0.02ᶠ | 2.82ᶜ |
|  | 0.05ᵃ | 0.00ᶠ | 0.04ᵃᵇᶜᵈ | 0.00ᵃᵇ | 3.10ᶜ | 0.00ᶠ | 0.01ᵃ | 0.01ᵃ | 0.53ᵃ | 0.47ᵃ | 0.45ᵃᵇ | 0.09ᵃ | 0.59ᵃ | 0.02ᵃ | 0.04ᵃ | 0.01ʰᶦʲᵏ | 0.00ᵇ | 0.00ᶜ | 0.01ᶠ | 0.14ʲᵏ |
